# Non-Malignant Respiratory Illnesses in Association with Occupational Exposure to Asbestos and Other Insulating Materials: Findings from the Alberta Insulator Cohort

**DOI:** 10.3390/ijerph17197085

**Published:** 2020-09-28

**Authors:** Subhabrata Moitra, Ali Farshchi Tabrizi, Kawtar Idrissi Machichi, Samineh Kamravaei, Noushin Miandashti, Linda Henderson, Manali Mukherjee, Fadi Khadour, Muhammad T. Naseem, Paige Lacy, Lyle Melenka

**Affiliations:** 1 Alberta Respiratory Centre, Department of Medicine, University of Alberta, Edmonton, AB T6G 2R3, Canada; moitra@ualberta.ca (S.M.); afarshch@ualberta.ca (A.F.T.); kawtar.idrissi@ualberta.ca (K.I.M.); kamravae@ualberta.ca (S.K.); miandash@ualberta.ca (N.M.); l.melenka@synergyrespiratory.com (L.M.); 2 Synergy Respiratory & Cardiac Care, Sherwood Park, AB T8H 0N2, Canada; l.henderson@synergyrespiratory.com (L.H.); fkhadour@ualberta.ca (F.K.); naseem@ualberta.ca (M.T.N.); 3 Department of Medicine, McMaster University & Firestone Institute for Respiratory Health, St. Joseph’s Healthcare, Hamilton, ON L8N 4A6, Canada; mukherj@mcmaster.ca

**Keywords:** insulation materials, inhaled particles, asbestos, pulmonary function tests, occupational health

## Abstract

Many insulating materials are used in construction, although few have been reported to cause non-malignant respiratory illnesses. We aimed to investigate associations between exposures to insulating materials and non-malignant respiratory illnesses in insulators. In this cross-sectional study, 990 insulators (45 ± 14 years) were screened from 2011–2017 in Alberta. All participants underwent pulmonary function tests and chest radiography. Demographics, work history, and history of chest infections were obtained through questionnaires. Chronic obstructive pulmonary disease (COPD) was diagnosed according to established guidelines. Associations between exposures and respiratory illnesses were assessed by modified Poisson regression. Of those screened, 875 (88%) were males. 457 (46%) participants reported having ≥ 1 chest infection in the past 3 years, while 156 (16%) were diagnosed with COPD. In multivariate models, all materials (asbestos, calcium silicate, carbon fibers, fiberglass, and refractory ceramic fibers) except aerogels and mineral fibers were associated with recurrent chest infections (prevalence ratio [PR] range: 1.18–1.42). Only asbestos was associated with COPD (PR: 1.44; 95% confidence interval [CI]: 1.01, 2.05). Therefore, occupational exposure to insulating materials was associated with non-malignant respiratory illnesses, specifically, recurrent chest infections and COPD. Longitudinal studies are urgently needed to assess the risk of exposure to these newly implemented insulation materials.

## 1. Introduction 

Insulators are engaged in the application, removal, and repair of thermal protective shields in buildings, pipelines, and industrial settings. In Alberta, a province of Canada, there are ~4100 unionized insulators [1] who are exposed to a wide variety of substances including, but not limited to, asbestos, calcium silicate, fiberglass, and other man-made vitreous fibers (MMVFs). One of the potential health hazards associated with this occupation is the risk of inhalation of hazardous substances that are known to have systemic and target organ effects, particularly the respiratory system [2]. Nevertheless, due to a rapid expansion in population, consumer demands, and innovative new materials, many new substances are being introduced in insulation, the potential risks of many of which remain unknown [2,3]. 

Asbestos has remained one of the most in-demand substances used in insulation in the world for many decades, primarily in the construction sector [2,3]. Despite the initial ban of chrysotile asbestos mining and production in Canada in the late 90s, chrysotile and other mineral forms of asbestos, such as actinolite, anthophyllite, tremolite, amosite, and crocidolite were still imported and used, particularly for insulation purposes, until as recently as 2018 [4,5]. In 2017, Canada imported 65 tonnes of asbestos [4]. Although respiratory illnesses associated with asbestos exposure have been investigated extensively, the majority of literature focused on asbestos-associated malignant conditions, specifically mesothelioma [6,7,8,9]. Evidence for the association of asbestos with other non-malignant respiratory conditions such as chronic obstructive pulmonary disease (COPD) and asthma is limited, and findings are inconsistent. While some studies showed a decline in lung function [10] and airway obstruction [11,12] associated with asbestos exposure, other reports do not confirm these associations and instead argue that this was a probable effect of smoking [13,14,15]. Moreover, despite a well-established relationship between asbestos and aggravated immunomodulation in the lungs [16], the association between asbestos exposure and the risk of pulmonary illnesses and concomitant infection has not been systematically investigated.

In addition to asbestos, several newer man-made materials such as aerogels, carbon fibers, mineral fibers, and refractory ceramic fibers have been introduced as alternative insulating materials. Aerogels are primarily made of silica [17]; however, they can also be prepared from natural sources and are commonly known as cellulose nanofiber biocomposite (CNF) aerogels [18]. Aerogels are synthetic, porous, ultralight materials with extremely low density and are very poor conductors of heat, which make these ideal for insulation. However, apart from the only available safety data sheet from the National Institute of Occupational Safety and Health (NIOSH) [19], information on health-related issues due to occupational exposure to aerogels is not available. Among other MMVFs, mineral fibers and refractory ceramic fibers have been identified as potential respiratory hazards, and their inhalation may lead to pulmonary carcinoma [20], adverse pleural conditions [21,22], and to some extent, interstitial changes in the lungs [23]. However, whether these MMVFs are associated with any chronic respiratory illnesses has not been systematically investigated.

In this study, we aim to investigate the relationship between occupational exposures to different insulating materials in use (asbestos, aerogels, calcium silicate, carbon fibers, fiberglass, mineral fibers, and refractory ceramic fibers) and respiratory conditions (chest infections, COPD and asthma) in a cohort of insulators.

## 2. Methodologies

### 2.1. Design and Participants

In this cross-sectional study, we investigated a cohort of unionized insulators belonging to the Local 110 Heat and Frost Insulators and Allied Workers Union in Edmonton, Alberta, who joined the Wellness of Workers (WoW) voluntary surveillance program in Alberta between 2011 and 2017. Workers were initially contacted by postal mail, emails, phone calls, brochures, and posters advertised on the Local 110 website by the WoW program. All 1043 insulators who registered with the Workers Union and participated in this study were screened at the section of occupational and environmental medicine at Synergy Respiratory Care Clinic, Sherwood Park, Alberta. Inclusion criteria were that participants were at least 18 years of age, employed full time as insulators, and of sound mental and cognitive ability at the time of screening. Exclusion criteria were individuals less than 18 years of age and trainees that did not have a history of insulation material exposure at the time of screening (*n* = 53 for the latter category). This study was approved by the Health Research Ethics Board of Alberta (HREBA.CTC-17-0067) and Health Research Ethics Board (Pro00079792), University of Alberta, and all participants provided signed informed consent forms before taking part in the study.

### 2.2. Questionnaire Assessment

The questionnaire used in this study was modified from the National Health and Nutrition Examination Survey (NHANES)-III [24], containing items of demographic profile (age, sex, smoking history, and frequency of smoking), personal and family history (exposure to smoke at childhood, second-hand smoke exposure, parental lung disease, family history of cancer, and having any allergies), and detailed job-exposure history including experience (years) and types of exposure to insulating materials (such as aerogels, asbestos, calcium silicate, carbon fibers, fiberglass, mineral fibers, and refractory ceramic fibers [RCFs]) as well as the use of personal protection equipment (PPE) at work. Use of PPE at workplace was dichotomized as those used it all the times (coded 0) and those who never used it or used infrequently (coded 1). The questionnaire was administered to all participants through an interviewer at the clinic.

Chest infection was self-reported and was considered if the participants gave positive responses to two consecutive questions: (i) “If you get a cold, does it usually (more than half of the time) go to your chest?” and (ii) “During the past three years, did you produce phlegm (mucus) with any of the chest illnesses that kept you off the work, at home, or in bed for a week or more?” Furthermore, responses to another question, “In the past three years, how many such illnesses with phlegm did you have which lasted a week or more?” were considered as the number (frequency) of chest infections in the past three years. 

### 2.3. Clinical Assessments

Pre and postbronchodilator spirometry was performed using a Vmax^®^ Encore pulmonary function test system (Vyaire Medical, Mettawa, IL, USA) according to the American Thoracic Society/European Respiratory Society guidelines for spirometry [25]. Reference values of spirometric indices were calculated from the Canadian Cohort of Obstructive Lung Diseases (CanCOLD) [26], percentage predicted values of forced expiratory volume in 1 s (FEV_1_ % predicted), and forced vital capacity (FVC % predicted) and their ratio (FEV_1_/FVC) were considered for this study. Participants were diagnosed to have asthma based on a diagnosis at the clinic, self-reported or physician-diagnosed asthma, and/or those with bronchodilator reversibility indicated by a change in FEV_1_ threshold of 12% and 200 mL. COPD was diagnosed if the participants had postbronchodilator FEV_1_/FVC < 70% [27]. 

### 2.4. Statistical Analyses

#### 2.4.1. Main Analyses

To test the relationship between each of the exposures and respiratory conditions (chest infections, COPD, and asthma), we first assessed the bivariate relationship between each of the exposure variables (aerogels, asbestos, calcium silicate, carbon, fiberglass, mineral fiber, and refractory ceramic fiber) and respiratory conditions (chest infections, COPD, and asthma) by χ^2^ test. Second, we constructed univariable (unadjusted) and multivariable (adjusted) regression models between exposures and each respiratory condition using modified Poisson regression models for binary outcomes with robust standard error [28]. Age, sex, body mass index (BMI), ethnicity, education, marital status, job history (years), smoking status (never, former/current), and smoking pack-years were tested as potential confounders. We used step-forward and -backward algorithms to build the models, and confounders were retained in the final model if: (i) they demonstrated significant association with both exposures and outcomes in the corresponding bivariate analyses or (ii) modified the estimates of the remaining variables in the main model by more than 10%. Model selection criteria included Pearson’s goodness-of-fit test, McFadden’s adjusted R^2^, and Akaike’s information criteria (AIC).

#### 2.4.2. Secondary Analyses

We also performed several secondary analyses. Firstly, we divided age into 4 quartiles and tested the association between exposures and respiratory conditions separately in each quartile. Secondly, we created negative binomial regression models to test the associations between the exposures and number of chest infections in the past 3 years. Thirdly, we additionally adjusted the single-exposure Poisson models by the use of PPE for the corresponding exposures (information was available for aerogels, asbestos, fiberglass, and mineral fiber only). Fourthly, we created multi-exposure Poisson models for each of the respiratory conditions, i.e., adding all exposures in the same model if the exposure variables did not demonstrate collinearity among them (variance inflation factor, VIF < 3). Lastly, we tested potential effect modification by smoking (never smoker vs. current/former smoker), parental lung disease, exposure to smoke at childhood, family history of cancer, and having any allergies. All analyses were conducted using a complete case approach in Stata V.15.1 (StataCorp, College Station, TX, USA), and a *p*-value < 0.05 was considered as statistically significant. The present study is compliant with the Strengthening the Reporting of Observational Studies in Epidemiology (STROBE) guidelines [29].

## 3. Results

### 3.1. Characteristics of the Study Population

Most participants were male (88%) with a mean age of 45 (standard deviation, SD: 14) years. 34% were never-smokers and had a median experience (job history) of 13 (interquartile range, IQR: 4, 29) years (Table 1). Mean (SD) postbronchodilator FEV_1_ (% predicted) was 94 (16). Exposure to asbestos, aerogels, calcium silicate, carbon, fiberglass, mineral fiber, and refractory ceramic fiber was reported by 55, 40, 89, 60, 86, 96, and 64% insulators, respectively. A total of 457 (46%) participants reported having at least one chest infection in the last 3 years, whereas COPD and asthma were clinically diagnosed among 16% and 4% of the participants, respectively. 

### 3.2. Association between Exposure and Respiratory Conditions

**Figure 1:**
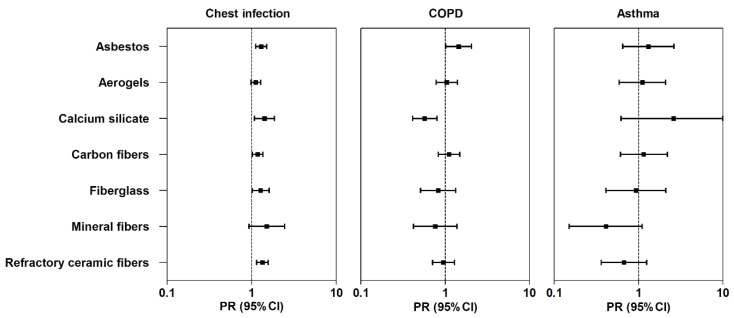
Association between different occupational exposures at work and respiratory conditions in insulators. Figure legend: adjusted associations between occupational exposures and respiratory conditions in insulators. Boxes and the error bars indicate the prevalence ratios (PR) and 95% confidence intervals (95%CI), respectively. Multivariable models were adjusted for sex, age, education (3 categories: up to high school, trade school, and college or beyond), and smoking pack-years. Each row represents a single regression model.

In bivariate analyses, we found that asbestos, calcium silicate, carbon fibers, fiberglass, and refractory ceramic fibers were significantly associated with chest infections, while mineral fiber exposure showed only marginal association. Aerogels were not associated with chest infections. Only asbestos was found to be associated with COPD, while none of the exposures were associated with asthma. In single exposure multivariable models (Figure 1), we found that all exposures, except aerogels and mineral fibers, were significantly associated with a higher prevalence of chest infections, whereas exposure to mineral fibers was marginally associated with chest infections. We also observed similar association between exposures and frequency of chest infections in negative binomial models (Table S1). In single exposure models, while asbestos was significantly associated with COPD (prevalence ratio [PR]: 1.45; 95% confidence interval [CI]: 1.01, 2.05), an inverse association was observed between exposure to calcium silicate and COPD (PR: 0.57; 9%%CI: 0.41, 0.80). However, upon stratifying the analysis by quartiles of age, we found an inverse association between calcium silicate and COPD (PR: 0.53; 95%CI: 0.34, 0.83) in the 4th quartile due to relatively fewer numbers of COPD cases among those exposed to calcium silicate than the three other quartiles (Q1–Q3) grouped together (PR: 0.68; 95%CI: 0.41, 1.13); thus, the association tended towards inverse in the pooled data. We did not observe any association between occupational exposures and asthma. In secondary analyses after additional adjustment for PPE, only asbestos was found to be consistently associated with chest infections (Table S2) and COPD (Table S3), but no association was observed in asthma (Table S4).

### 3.3. Sensitivity Analyses

In multi-exposure models, we did not observe any collinearity between the exposures (VIF < 3), and only asbestos and refractory ceramic fibers remained associated with chest infections (Table S5). Although compared to single exposure models, the magnitude of associations lowered marginally, and the precision remained consistent (*p* < 0.05). The association between asbestos exposure and COPD persisted in the multi-exposure model, although the magnitude of the estimate increased moderately in the multi-exposure model (PR: 1.55; 95%CI: 1.08, 2.22) compared to the single-exposure model. While exposure to mineral fibers was found inversely associated with asthma in the multi-exposure model (PR: 0.22; 95%CI: 0.06, 0.80), the association was inconsistent compared to the single exposure model. 

Interestingly, although the probability of chest infections due to exposures (except aerogels and asbestos) trended to higher levels in current smokers, those differences were not statistically significant (Table S6). We did not observe any effect modification by smoking on the association between exposures and COPD or asthma (Table S6). We did not observe any effect modification by parental lung disease, exposure to smoke at childhood, and family history of cancer (Tables S7–S9). Although presence of any allergies had a significant (*p* = 0.01) influence on the association between fiberglass exposure and COPD, i.e., a higher association between fiberglass and COPD among the nonallergic individuals (PR: 1.93; 95%CI: 0.74, 5.03) than those who were allergic (PR: 0.48; 9%%CI: 0.27, 0.87) (Table S10), such influence was not found on other associations between exposures and respiratory illnesses.

## 4. Discussion

### 4.1. Interpretation of Results in Context of Available Evidence

To our knowledge, this is the first report of a cohort assessing the relationship between several types of insulating materials and non-malignant respiratory conditions among insulators. In this study, we observed that all insulating materials, except aerogels and mineral fibers, were associated with an increased probability of chest infections, while only asbestos exposure was marginally associated with higher prevalence of COPD. No association was found between insulating materials and the prevalence of asthma.

In our study, asbestos was significantly associated with a higher prevalence of repeated chest infections. Although literature emphasizing chest infections as a result of asbestos exposure is scanty, it has been reported that exposure to asbestos may increase the risk of pneumonia [30,31]. We found asbestos to be associated with COPD both in single and multi-exposure models. Our observations largely resonate with previous findings where progressive lung function decline and COPD have been reported in workers with history of asbestos exposure [10,12,32,33]. The significant association between asbestos and COPD in a multi-exposure model (as we did not observe any collinearity between exposures, VIF < 3) can also be explained by the fact that asbestos may cause airflow limitation in the presence of other materials, vapors, gases, or dusts, as occurs in the real world of insulating material application [34]. However, more clinical epidemiological studies, especially longitudinal studies, are required to understand the relationship between asbestos exposure and chronic airway disease. Human biomonitoring could be applied through specific biomarkers of exposure and/or detection of early effects to assess potential health risks associated with recent exposure to insulating materials including asbestos.

The association between aerogels and all respiratory conditions towards null is plausible since aerogels appear to impose lesser health risks than any other materials used in insulation. Although the Occupational Safety and Health Agency (OSHA) described aerogels as a plausible irritant of the skin and upper airways [19], epidemiological and mechanistic studies on the plausible health effects of aerogels are scarce. Conversely, hybrid organic–inorganic aerogels are now being considered as a promising agent for targeted drug delivery, wound healing, and bone regeneration [35,36,37]. Therefore, whether aerogels impart beneficial or detrimental health effects needs to be understood through properly designed experimental and epidemiological research.

We also observed a significant association between exposure to calcium silicate and recurrent chest infections. Although evidence on whether calcium silicate as a standalone exposure is associated with respiratory damage is not well reported, it has been shown that calcium silicate is the principal component of wollastonite, a naturally occurring mineral often used as building and plastic industries [38], and silicate fibers of wollastonite might impose respiratory hazards [38]. Wollastonite has been reported to accelerate inflammatory responses in alveolar macrophages and pneumocytes, both *in vivo* and *in vitro* [39], and presumably increases the risk of infection. However, despite a plausible link between calcium silicate and the risk of fibrosis, an association of calcium silicate with airway obstruction has not been reported yet. While we observed an inverse association between calcium silicate exposure and COPD, this association did not persist after excluding those in the 4th quartile of age, as discussed above. An earlier study showed lung function decline among wollastonite-exposed workers who developed fibrosis that was greater than those who did not have fibrotic plaques [40]. However, any association of calcium silicate exposure with lung function decline has not been demonstrated so far. Future cohorts should be considered to assess the long-term respiratory impact of calcium silicate exposure.

Similar to asbestos and calcium silicate, we also observed significant association between exposure to carbon fibers and recurrent chest infections. This is a novel finding as engineered carbon fibers used in the insulation process have not been reported to cause any respiratory illnesses to date. However, one potential mode by which respiratory illnesses could occur may be through the generation of carbon nanotubes (CNTs) during processing of those fibers, and CNTs have been reported to increase susceptibility to microbial infections in the respiratory tract [41,42]. One animal study has shown that exposure to CNTs may impede with bacterial clearance from lungs and also increase the risk of infection [43], while another has shown alteration of pulmonary macrophage function due to CNTs in experimental COPD models [44]. However, no human studies on this topic have been reported. It must be remembered that exposure to carbon fibers may not necessarily be equivalent to CNT exposure, so results from animal studies should be interpreted cautiously. 

We found a strong association between exposure to fiberglass and repetitive chest infections, and there was also a clinically important association between mineral fibers and chest infections, albeit the association was not statistically significant. Several articles have described potential health effects of mineral fibers [23,45,46,47]; however, only a handful number of reports clearly described its relationship with chest infections [48]. It has been observed that development of pneumonia among workers with a history of mineral fiber exposure could be a direct consequence of such exposure [31], as these fibers impart detrimental effects on the natural functioning of the airway macrophages [49]. Most of the studies related to mineral fibers have focused on fibrosis and other interstitial consequences, whereas their association with lung function or airway diseases have mostly remained unearthed. We found no association between fiberglass/mineral fibers and COPD or asthma, which is in line with the findings of the only available study where an association between fiberglass exposure and FEV_1_ was found in a group of workers that maintained normal lung function, and no association was found between fiberglass and COPD or asthma [21]. More longitudinal occupational exposure studies are required to explore plausible effects of exposure to various mineral fibers on lung function changes.

Moreover, in the case of RCF exposure, we determined a significant association with recurrent chest infections. This is possibly because of the deleterious effects of man-made vitreous fibers (MMVFs) on the immune cells of the lungs [49], thus making lungs prone to infections. However, no epidemiological or mechanistic studies are available that might help us understand the link between RCFs and chest infections. We did not observe any association between RCFs and COPD or asthma in our study. Association between RCFs and lung function decline has been mixed in previous results; however, a majority of the studies did not observe any clinically significant decline in lung function in RCF-exposed workers [50,51], and those that reported RCF-associated decline in lung function were either influenced by smoking [52], high cumulative exposure [53], or both [52]. Nevertheless, no study has reported any link between RCFs and clinically confirmed airway diseases, such as COPD and asthma. 

### 4.2. Clinical and Public Health Implications

Our findings provide an important public health message about insulating material-associated respiratory conditions, and we anticipate that these results will help workers, and associated labour committees, to comprehend plausible health consequences due to exposure to such materials. Our observations will also aid public health experts in recommending suitable preventive measures for insulators, such as choosing less hazardous substances like aerogels, and ensuring consistent use of PPE while at work. Moreover, we also demonstrated a higher prevalence of chest infections and COPD in insulators than in the average general population in Canada [54,55], which indicates a plausible detrimental effect of occupational exposure to insulating materials on the respiratory health of the workers. These results strongly vouch for an immediate surveillance of the labour policymakers to reduce the use of hazardous insulating materials as a general policy measure to minimize the burden of work-related health hazards among insulators. 

### 4.3. Strengths and Limitations

One of the major strengths of this study lies in its approach in considering a wide range of materials used in insulation that helps in comprehending the exposure scenario of workers. Secondly, we performed several secondary analyses to test the plausible involvement of any other factors in the association between exposures and respiratory illnesses, which substantiate the robustness of our analytical methods and findings. 

The limitations of this study should also be kept in mind. This is a cross-sectional study; therefore, we could not determine any causal association. However, our results are substantiated by previous epidemiological evidence, particularly related to asbestos and refractory ceramic fibers in association with respiratory complications. Despite taking into account a wide variety of insulating materials, we could not measure actual workplace exposures. Secondly, we could not measure the level of exposure to each insulating material, and participants were also unable to recollect the exact duration of exposure to each of these materials over many years; therefore, a cumulative exposure index was not achievable. Moreover, an assessment of individual exposures separately could have provided more information about their potential toxicity. However, the application of material-specific experiments would be impossible to carry out in occupational exposure studies, particularly where workers are exposed to a wide range of materials in their work. Information about PPEs was inadequate; thus, we could not assess the efficacy of PPE usage. However, based on our analyses (see Tables S2–S4), it can be assumed that the PPEs employed by workers, which may have been inconsistently used, did not provide substantial protection for workers from exposures. Moreover, information about residual confounding, such as other plausible workplace exposures including vapor, dust, gas and fumes (VDGFs), physical and chemical agents, or socioenvironmental triggers (such as indoor conditions of the workers, environmental pollen concentration, among others), were not available and need to be considered in future studies.

## 5. Conclusions

We conclude that chronic occupational exposure to insulation materials in insulators is associated with an increased prevalence of chest infections and, to a lesser degree, COPD. Adaptation of less hazardous insulating materials such as aerogels, and proper use of PPE would be beneficial to minimize the burden of work-related lung diseases. An immediate workplace surveillance program must be initiated to characterize these exposures, and necessary measures to protect the insulators against exposure to hazardous materials as well as regular health monitoring of the workers are warranted.

## Figures and Tables

**Table 1:** Demographic characteristics, exposure history, and clinical profiles of the insulators.

	*N* = 990
**Demographics**	
Sex (male), *n* (%)	875 (88)
Age (years), mean (SD)	45 (14)
1st quartile (*n* = 239)	28 (4)
2nd quartile (*n* = 237)	39 (4)
3rd quartile (*n* = 256)	51 (3)
4th quartile (*n* = 258)	62 (6)
BMI (kg/m^2^), mean (SD)	29.3 (5.5)
Ethnicity, *n* (%)	
Caucasian	792 (80)
African/Afro-American	63 (6)
Asian/Middle-Eastern	38 (4)
Hispanic	65 (7)
Aboriginal	32 (3)
Education, *n* (%)	
Up to high school	163 (16)
Trade school	490 (50)
College or beyond	337 (34)
Marital status, *n* (%)	
Married/common law-partner	592 (60)
Smoking history	
Never smokers, *n* (%)	332 (34)
Overall pack-years, median (IQR)	4.7 (0, 18)
Job history (years), median (IQR)	13 (4, 29)
Passively smoke exposure at childhood, *n* (%)	661 (67)
Parental lung disease, *n* (%)	250 (25)
Family history of cancer, *n* (%)	285 (29)
**Clinical features**	
Allergies, *n* (%)	
Any allergies	416 (42)
Hay fever ^†^	111 (11)
Eczema ^†^	46 (5)
Hives ^†^	36 (4)
Other allergies ^†^	340 (34)
FEV_1_ (% predicted), mean (SD)	94.0 (15.8)
FVC (% predicted), mean (SD)	100.3 (15.1)
FEV_1_/FVC, mean (SD)	76.7 (8.5)
**Exposure details**	
Ever exposed to asbestos, *n* (%)	547 (55)
Ever exposed to aerogel, *n* (%)	392 (40)
Ever exposed to calcium silicate, *n* (%)	877 (89)
Ever exposed to carbon fibers, *n* (%)	597 (60)
Ever exposed to fiberglass, *n* (%)	849 (86)
Ever exposed to mineral fibers, *n* (%)	950 (96)
Ever exposed to refractory ceramic fibers, *n* (%)	632 (64)
Use of PPE for	
Asbestos, *n* (%)	276 (28)
Aerogels, *n* (%)	303 (31)
Calcium silicate, *n* (%)	NA
Carbon fibers, *n* (%)	NA
Fiberglass, *n* (%)	105 (11)
Mineral fibers, *n* (%)	148 (15)
Refractory ceramic fibers, *n* (%)	NA
**Respiratory conditions**	
Having a chest infection within past 3 years, *n* (%)	457 (46)
Frequency of chest infection within past 3 years, median (IQR) ^‡^	2 (1, 3)
COPD, *n* (%)	156 (16)
Asthma, *n* (%)	40 (4)

Data presented as frequency (%), mean (standard deviation: SD), or median (interquartile range: IQR), unless otherwise specified. ^†^ these different allergies do not add up to 100% (any allergies) due to multiple nonexclusive response. ^‡^ those who had a chest infection at least once in the past 3 years (*n* = 457). Abbreviations: BMI: body mass index; COPD: chronic obstructive pulmonary disease; FEV_1_: forced expiratory volume in 1 s; FVC: forced vital capacity; PPE: personal protective equipment; and NA: data not available.

## Supplementary Materials

The following are available online at https://www.mdpi.com/1660-4601/17/19/7085/s1, Table S1: Association between different occupational exposures at work and frequency of chest infections in the last 3 years in insulators, Table S2: Association between different occupational exposures at work and chest infection in insulators, Table S3: Association between different occupational exposures at work and COPD in insulators, Table S4: Association between different occupational exposures at work and asthma in insulators, Table S5: Association between different occupational exposures at work (multi-exposure models) and respiratory conditions in insulators, Table S6: Effect modification by smoking on the association between different occupational exposures at work and respiratory conditions in insulators, Table S7: Effect modification by exposure to smoke at childhood on the association between different occupational exposures at work and respiratory conditions in insulators, Table S8: Effect modification by parental lung disease on the association between different occupational exposures at work and respiratory conditions in insulators, Table S9: Effect modification by family history of cancer on the association between different occupational exposures at work and respiratory conditions in insulators, Table S10: Effect modification by any allergies on the association between different occupational exposures at work and respiratory conditions.

## Data Sharing Policy

Data of this manuscript may contain sensitive personal information, and share, transfer, or modification of the data is prohibited by law. However, an anonymous dataset with limited information can be shared upon reasonable request made to the corresponding author.
