# Supplementary material for: Non-Malignant Respiratory Illnesses in Association with Occupational Exposure to Asbestos and Other Insulating Materials: Findings from the Alberta Insulator Cohort
Source: Int J Environ Res Public Health. 2020 Sep 28;17(19):7085. doi: 10.3390/ijerph17197085 (PMC7579178; doi:10.3390/ijerph17197085)
Supplement: Supplementary file 1 [file ijerph-17-07085-s001.pdf]

Non-malignant Respiratory Illnesses in Association with Occupational Exposure to Asbestos and Other Insulating Materials: Findings from the Alberta Insulator Cohort

Subhabrata Moitra, Ali Farshchi Tabrizi, Kawtar Idrissi Machichi, Samineh Kamravaei, Noushin Miandashti, Linda Henderson, Manali Mukherjee, Fadi Khadour, Muhammad T. Naseem, Paige Lacy, Lyle Melenka

List of supplementary Tables:

Supplementary Table S1: Association between different occupational exposures at work and frequency of chest infections in the last 3 years in insulators.

Supplementary Table S2: Association between different occupational exposures at work and chest infection in insulators.

Supplementary Table S3: Association between different occupational exposures at work and COPD in insulators.

Supplementary Table S4: Association between different occupational exposures at work and asthma in insulators.

Supplementary Table S5: Association between different occupational exposures at work (multi-exposure models) and respiratory conditions in insulators.

Supplementary Table S6: Effect modification by smoking on the association between different occupational exposures at work and respiratory conditions in insulators.

Supplementary Table S7: Effect modification by exposure to smoke at childhood on the association between different occupational exposures at work and respiratory conditions in insulators.

Supplementary Table S8: Effect modification by parental lung disease on the association between different occupational exposures at work and respiratory conditions in insulators.

Supplementary Table S9: Effect modification by family history of cancer on the association between different occupational exposures at work and respiratory conditions in insulators.

Supplementary Table S10: Effect modification by any allergies on the association between different occupational exposures at work and respiratory conditions.

Supplementary Table S1: Association between different occupational exposures at work and frequency of chest infections in the past 3 years in insulators.

Exposures	Frequency of chest infection β (95%CI) Unadjusted	Adjusted
Asbestos	**0.35 (0.14, 0.56)**	**0.50 (0.26, 0.73)**
Aerogels	-0.03 (-0.25, 0.18)	-0.02 (-0.23, 0.19)
Calcium silicate	**0.68 (0.32, 1.04)**	**0.73 (0.37, 1.09)**
Carbon fibers	**0.31 (0.09, 0.52)**	**0.30 (0.09, 0.51)**
Fiberglass	**0.59 (0.28, 0.91)**	**0.59 (0.27, 0.91)**
Mineral fibers	**0.85 (0.23, 1.46)**	**0.73 (0.13, 1.34)**
Refractory ceramic fibers	**0.68 (0.46, 0.90)**	**0.64 (0.43, 0.86)**

Data shown as regression coefficient (β) and 95% confidence interval (95%CI) obtained from negative binomial regression models taking frequencies of chest infections in the past 3 years as a count variable. In adjusted modes, sex, age, education (3 categories: up to high school, trade school and college or beyond), and smoking pack-years were considered as confounders. Each row is a single exposure model.

Supplementary Table S2: Association between different occupational exposures at work and chest infection in insulators.

**Exposures**	**Chest infection**		
	**PR (95%CI)**		
	**Model-1**	**Model-2**	**Model-3**
Asbestos	**1.29 (1.12, 1.49)**	**1.30 (1.12, 1.51)**	**1.42 (1.18, 1.72)**
Aerogels	1.11 (0.97, 1.27)	1.12 (0.98, 1.28)	0.89 (0.63, 1.26)
Calcium silicate	**1.41 (1.08, 1.84)**	**1.42 (1.08, 1.86)**	NA
Carbon fibers	**1.19 (1.03, 1.37)**	**1.18 (1.02, 1.36)**	NA
Fiberglass	**1.32 (1.05, 1.67)**	**1.27 (1.01, 1.61)**	1.28 (1.00, 1.64)
Mineral fibers	1.56 (0.97, 2.52)	1.51 (0.93, 2.45)	1.47 (0.89, 2.43)
Refractory ceramic fibers	**1.38 (1.18, 1.61)**	**1.34 (1.15, 1.57)**	NA

Data shown as prevalence ratio (PR) and 95% confidence interval (95%CI) obtained from modified Poisson regression models. Model-1: unadjusted models; Model-2: adjusted for sex, age, education (3 categories: up to high school, trade school and college or beyond), and smoking pack-years. Model-3: Model-2 + additional adjustment for personal protective equipments (PPE) for each of the exposures. Each row is a single exposure model. NA: PPE information not available for those exposures.

Supplementary Table S3: Association between different occupational exposures at work and COPD in insulators.

**Exposures**	**COPD**		
	**PR (95%CI)**		
	**Model-1**	**Model-2**	**Model-3**
Asbestos	**2.70 (1.90, 3.83)**	**1.44 (1.01, 2.05)**	**1.52 (1.03, 2.24)**
Aerogels	0.85 (0.63, 1.16)	1.04 (0.78, 1.39)	1.10 (0.67, 1.81)
Calcium silicate	0.70 (0.48, 1.04)	**0.57 (0.41, 0.80)**	NA
Carbon fibers	1.28 (0.94, 1.74)	1.11 (0.83, 1.49)	NA
Fiberglass	1.45 (0.89, 2.36)	0.83 (0.51, 1.33)	0.89 (0.54, 1.47)
Mineral fibers	0.90 (0.45, 1.79)	0.76 (0.42, 1.38)	0.82 (0.43, 1.55)
Refractory ceramic fibers	1.20 (0.88, 1.63)	0.95 (0.71, 1.29)	NA

Data shown as prevalence ratio (PR) and 95% confidence interval (95%CI) obtained from modified Poisson regression models. Model-1: unadjusted models; Model-2: adjusted for sex, age, education (3 categories: up to high school, trade school and college or beyond), and smoking pack-years. Model-3: Model-2 + additional adjustment for personal protective equipments (PPE) for each of the exposures. Each row is a single exposure model. NA: PPE information not available for those exposures.

Supplementary Table S4: Association between different occupational exposures at work and asthma in insulators.

Exposures	Asthma PR (95%CI)		
	Model-1	Model-2	Model-3
Asbestos	0.99 (0.54, 1.82)	1.31 (0.65, 2.63)	1.36 (0.97, 1.90)
Aerogels	1.13 (0.61, 2.08)	1.11 (0.59, 2.09)	1.16 (0.28, 4.78)
Calcium silicate	2.43 (0.59, 9.93)	2.59 (0.62, 9.98)	NA
Carbon fibers	1.10 (0.59, 2.06)	1.15 (0.61, 2.20)	NA
Fiberglass	0.78 (0.35, 1.74)	0.93 (0.41, 2.11)	0.77 (0.29, 2.06)
Mineral fibers	0.38 (0.14, 1.01)	0.41 (0.15, 1.10)	**0.26 (0.07, 0.93)**
Refractory ceramic fibers	0.62 (0.34, 1.15)	0.67 (0.36, 1.25)	NA

Data shown as prevalence ratio (PR) and 95% confidence interval (95%CI) obtained from modified Poisson regression models. Model-1: unadjusted models; Model-2: adjusted for sex, age, education (3 categories: up to high school, trade school and college or beyond), and smoking pack-years. Model-3: Model-2 + additional adjustment for personal protective equipments (PPE) for each of the exposures. Each row is a single exposure model. NA: PPE information not available for those exposures.

Supplementary Table S5: Association between different occupational exposures at work (multiexposure models) and respiratory conditions in insulators.

**Exposures**	**Chest infection**		**COPD**		**Asthma**	
	**PR (95%CI)**		**PR (95%CI)**		**PR (95%CI)**	
	**Model-1**	**Model-2**	**Model-1**	**Model-2**	**Model-1**	**Model-2**
Asbestos	**1.19 (1.03, 1.38)**	**1.21 (1.04, 1.42)**	**2.79 (1.94, 4.05)**	**1.55 (1.08, 2.22)**	1.05 (0.54, 2.05)	**1.36 (0.64, 2.87)**
Aerogels	1.07 (0.94, 1.23)	1.08 (0.94, 1.23)	0.83 (0.62, 1.13)	1.05 (0.79, 1.39)	1.13 (0.62, 2.08)	1.09 (0.57, 2.06)
Calcium silicate	1.11 (0.83, 1.48)	1.15 (0.86, 1.55)	**0.49 (0.32, 0.76)**	**0.48 (0.32, 0.72)**	5.19 (0.90, 30.0)	5.04 (0.86, 29.6)
Carbon fibers	1.07 (0.92, 1.23)	1.07 (0.92, 1.24)	1.23 (0.89, 1.68)	1.21 (0.90, 1.62)	1.22 (0.62, 2.37)	1.20 (0.62, 2.34)
Fiberglass	1.09 (0.85, 1.40)	1.08 (0.84, 1.38)	1.10 (0.66, 1.83)	0.82 (0.49, 1.37)	0.93 (0.36, 2.40)	1.04 (0.41, 2.63)
Mineral fibers	1.17 (0.71, 1.92)	1.11 (0.67, 1.83)	0.88 (0.43, 1.80)	1.06 (0.52, 2.16)	**0.22 (0.06, 0.83)**	**0.22 (0.06, 0.80)**
Refractory ceramic fibers	**1.25 (1.06, 1.47)**	**1.22 (1.03, 1.44)**	1.15 (0.82, 1.63)	1.09 (0.78, 1.53)	0.54 (0.29, 1.02)	0.56 (0.30, 1.04)

Data shown as prevalence ratio (PR) and 95% confidence interval (95%CI) obtained from modified Poisson regression models. Model-1: multiexposure (all exposures) models without adjustment for confounders. Model-2: multiexposure (all exposures) models adjusted for sex, age, education (3 categories: up to high school, trade school and college or beyond), and smoking pack-years.

Supplementary Table S6: Effect modification by smoking on the association between different occupational exposures at work and respiratory conditions in insulators.

Exposures	Smoking history			Smoking history			Smoking history		
	NS (N=332)	S (N=658)		NS (N=332)	S (N=658)		NS (N=332)	S (N=658)	
	Chest infection			COPD			Asthma		
	PR (95%CI)	PR (95%CI)	#p	PR (95%CI)	PR (95%CI)	#p	PR (95%CI)	PR (95%CI)	#p
Asbestos	**1.57 (1.18, 2.11)**	**1.19 (1.00, 1.43)**	0.08	2.02 (0.82, 4.96)	1.35 (0.93, 1.96)	0.17	0.59 (0.18, 1.92)	2.03 (0.76, 5.44)	0.13
Aerogels	0.98 (0.68, 1.41)	0.98 (0.76, 1.26)	0.94	1.83 (0.80, 4.19)	0.93 (0.68, 1.26)	0.37	0.39 (0.11, 1.35)	1.77 (0.81, 3.85)	0.06
Calcium silicate	1.18 (0.70, 1.96)	**1.55 (1.13, 2.13)**	0.45	0.44 (0.17, 1.15)	**0.68 (0.46, 0.99)**	0.21	NE	1.82 (0.44, 7.61)	NE
Carbon fibers	0.97 (0.74, 1.28)	**1.28 (1.09, 1.52)**	0.12	1.06 (0.47, 2.41)	1.15 (0.85, 1.56)	0.81	1.45 (0.48, 4.42)	0.98 (0.44, 2.19)	0.56
Fiberglass	1.30 (0.87, 1.94)	1.26 (0.95, 1.66)	0.78	NE	0.84 (0.52, 1.35)	NE	3.60 (0.51, 25.3)	0.48 (0.20, 1.16)	0.07
Mineral fibers	1.37 (0.58, 3.24)	1.58 (0.89, 2.80)	0.85	1.61 (0.55, 4.72)	0.79 (0.42, 1.51)	0.72	0.26 (0.06, 1.06)	0.50 (0.13, 1.93)	0.60
Refractory ceramic fibers	1.18 (0.89, 1.58)	**1.44 (1.20, 1.73)**	0.28	0.89 (0.41, 1.96)	1.01 (0.74, 1.39)	0.80	0.70 (0.24, 2.04)	0.65 (0.31, 1.39)	0.99

Data shown as prevalence ratio (PR) and 95% confidence interval (95%CI) obtained from modified Poisson regression models. Each row is a single exposure model. All models adjusted for sex, age and education (3 categories: up to high school, trade school and college or beyond). #p: p value for interaction; NS: nonsmoker; S: current/former smoker. NE: could not be estimated due to very small number.

Supplementary Table S7: Effect modification by exposure to smoke at childhood on the association between different occupational exposures at work and respiratory conditions in insulators.

Exposures	Exposure to smoke at childhood			Exposure to smoke at childhood			Exposure to smoke at childhood		
	No (N=329)	Yes (N=661)		No (N=329)	Yes (N=661)		No (N=329)	Yes (N=661)	
	Chest infection			COPD			Asthma		
	PR (95%CI)	PR (95%CI)	#p	PR (95%CI)	PR (95%CI)	#p	PR (95%CI)	PR (95%CI)	#p
Asbestos	**1.52 (1.17, 1.98)**	1.20 (0.99, 1.44)	0.33	2.28 (1.01, 5.11)	1.27 (0.86, 1.86)	0.26	1.52 (0.57, 4.08)	1.12 (0.42, 2.99)	0.61
Aerogels	1.20 (0.95, 1.53)	1.08 (0.92, 1.27)	0.47	1.13 (0.58, 2.21)	0.99 (0.73, 1.36)	0.72	0.92 (0.35, 2.44)	1.26 (0.53, 2.97)	0.52
Calcium silicate	1.19 (0.75, 1.88)	**1.53 (1.10, 2.14)**	0.30	0.67 (0.26, 1.71)	**0.57 (0.39, 0.82)**	0.63	NE	1.47 (0.35, 6.24)	NE
Carbon fibers	1.19 (0.92, 1.54)	1.17 (0.98, 1.39)	0.96	0.95 (0.50, 1.81)	1.20 (0.87, 1.65)	0.67	1.51 (0.54, 4.21)	0.94 (0.41, 2.15)	0.44
Fiberglass	1.14 (0.79, 1.64)	**1.36 (1.00, 1.84**)	0.38	0.59 (0.24, 1.44)	0.91 (0.51, 1.63)	0.55	1.60 (0.36, 7.15)	0.68 (0.25, 1.80)	0.34
Mineral fibers	2.68 (0.74, 9.75)	1.25 (0.75, 2.11)	0.29	0.53 (0.15, 1.89)	0.88 (0.44, 1.75)	0.45	0.59 (0.08, 4.26)	**0.27 (0.09, 0.87)**	0.53
Refractory ceramic fibers	**1.56 (1.19, 2.06)**	**1.24 (1.03, 1.50)**	0.22	1.45 (0.71, 2.94)	0.84 (0.61, 1.15)	0.19	1.06 (0.43, 2.65)	0.50 (0.22, 1.14)	0.21

Data shown as prevalence ratio (PR) and 95% confidence interval (95%CI) obtained from modified Poisson regression models. Each row is a single exposure model. All models adjusted for sex, age, education (3 categories: up to high school, trade school and college or beyond) and smoking pack-years. #p: p value for interaction. NE: could not be estimated due to very small number.

Supplementary Table S8: Effect modification by parental lung disease on the association between different occupational exposures at work and respiratory conditions in insulators.

**Exposures**	**Parental lung disease**			**Parental lung disease**			**Parental lung disease**		
	**No (N=740)**	**Yes (N=250)**		**No (N=740)**	**Yes (N=250)**		**No (N=740)**	**Yes (N=250)**	
	**Chest infection**			**COPD**			**Asthma**		
	**PR (95%CI)**	**PR (95%CI)**	**#p**	**PR (95%CI)**	**PR (95%CI)**	**#p**	**PR (95%CI)**	**PR (95%CI)**	**#p**
Asbestos	**1.32 (1.10, 1.58)**	1.24 (0.92, 1.68)	0.46	**1.87 (1.16, 3.01)**	0.85 (0.51, 1.41)	**0.03**	1.27 (0.53, 3.04)	1.62 (0.47, 5.63)	0.97
Aerogels	1.10 (0.93, 1.28)	1.19 (0.93, 1.51)	0.46	0.94 (0.64, 1.37)	1.29 (0.85, 1.94)	0.14	1.03 (0.47, 2.28)	1.30 (0.49, 3.46)	0.70
Calcium silicate	**1.42 (1.03, 1.95)**	1.40 (0.85, 2.30)	0.98	**0.56 (0.37, 0.87)**	**0.57 (0.34, 0.95)**	0.92	3.38 (0.46, 25.0)	1.56 (0.21, 11.5)	0.57
Carbon fibers	**1.21 (1.02, 1.44)**	1.12 (0.86, 1.47)	0.57	1.14 (0.79, 1.64)	1.04 (0.65, 1.66)	0.75	0.84 (0.40, 1.80)	1.72 (0.54, 5.48)	0.33
Fiberglass	1.29 (0.98, 1.70)	1.21 (0.77, 1.90)	0.77	0.94 (0.49, 1.78)	0.66 (0.34, 1.30)	0.43	0.80 (0.30, 2.16)	1.23 (0.24, 6.23)	0.75
Mineral fibers	1.43 (0.80, 2.54)	1.72 (0.72, 4.08)	0.73	0.74 (0.33, 1.65)	0.89 (0.41, 1.92)	0.64	0.46 (0.12, 1.82)	0.32 (0.08, 1.28)	0.89
Refractory ceramic fibers	**1.29 (1.08, 1.55)**	**1.50 (1.08, 2.07)**	0.49	1.01 (0.69, 1.48)	0.80 (0.50, 1.26)	0.41	0.56 (0.26, 1.21)	0.77 (0.28, 2.10)	0.65

Data shown as prevalence ratio (PR) and 95% confidence interval (95%CI) obtained from modified Poisson regression models. Each row is a single exposure model.

All models adjusted for sex, age, education (3 categories: up to high school, trade school and college or beyond) and smoking pack-years. #p: p value for interaction.

Supplementary Table S9: Effect modification by family history of cancer on the association between different occupational exposures at work and respiratory conditions in insulators.

Exposures	Family history of cancer			Family history of cancer			Family history of cancer		
	**No (N=704)**	**Yes (N=285)**		**No (N=704)**	**Yes (N=285)**		**No (N=704)**	**Yes (N=285)**	
	**Chest infection**			**COPD**			**Asthma**		
	**PR (95%CI)**	**PR (95%CI)**	**#p**	**PR (95%CI)**	**PR (95%CI)**	**#p**	**PR (95%CI)**	**PR (95%CI)**	**#p**
Asbestos	**1.32 (1.10, 1.58)**	1.21 (0.91, 1.60)	0.31	1.42 (0.92, 2.19)	1.48 (0.80, 2.72)	0.69	1.77 (0.81, 3.88)	0.66 (0.17, 2.58)	0.21
Aerogels	**1.18 (1.00, 1.39)**	0.95 (0.75, 1.20)	0.35	1.00 (0.69, 1.44)	1.06 (0.66, 1.69)	0.58	0.94 (0.44, 1.98)	1.71 (0.56, 5.19)	0.43
Calcium silicate	1.25 (0.91, 1.71)	**1.77 (1.03, 3.02)**	0.24	0.64 (0.40, 1.02)	0.50 (0.30, 0.85)	0.44	1.71 (0.41, 7.08)	NE	NE
Carbon fibers	1.18 (0.99, 1.40)	1.11 (0.86, 1.44)	0.86	1.16 (0.81, 1.67)	1.05 (0.66, 1.72)	0.55	1.08 (0.51, 2.28)	1.41 (0.40, 5.06)	0.83
Fiberglass	1.14 (0.89, 1.48)	1.69 (0.95, 3.00)	0.19	0.75 (0.43, 1.30)	1.02 (0.38, 2.72)	0.80	0.98 (0.38, 2.53)	0.83 (0.17, 4.06)	0.84
Mineral fibers	1.49 (0.83, 2.68)	1.44 (0.60, 3.49)	0.92	0.89 (0.41, 1.97)	0.62 (0.23, 1.65)	0.56	0.56 (0.14, 2.23)	0.23 (0.05, 1.08)	0.43
Refractory ceramic fibers	**1.30 (1.08, 1.57)**	**1.43 (1.06, 1.92)**	0.58	0.98 (0.67, 1.43)	0.91 (0.56, 1.47)	0.75	0.76 (0.37, 1.59)	0.53 (0.16, 1.74)	0.53

Data shown as prevalence ratio (PR) and 95% confidence interval (95%CI) obtained from modified Poisson regression models. Each row is a single exposure model. All models adjusted for sex, age, education (3 categories: up to high school, trade school and college or beyond) and smoking pack-years. #p: p value for interaction. NE: could not be estimated due to very small number.

Supplementary Table S10: Effect modification by any allergies on the association between different occupational exposures at work and respiratory conditions.

Exposures	Any allergies			Any allergies			Any allergies		
	**No (N=574)**	**Yes (N=416)**		**No (N=605)**	**Yes (N=438)**		**No (N=605)**	**Yes (N=438)**	
	**Chest infection**			**COPD**			**Asthma**		
	**PR (95%CI)**	**PR (95%CI)**	**#p**	**PR (95%CI)**	**PR (95%CI)**	**#p**	**PR (95%CI)**	**PR (95%CI)**	**#p**
Asbestos	**1.36 (1.10, 1.68)**	1.21 (0.97, 1.52)	0.19	**1.75 (1.10, 2.79)**	0.95 (0.54, 1.69)	0.14	2.10 (0.62, 7.10)	0.89 (0.35, 2.27)	0.18
Aerogels	**1.25 (1.04, 1.50)**	0.97 (0.80, 1.19)	0.14	1.00 (0.69, 1.45)	1.11 (0.72, 1.70)	0.74	1.15 (0.42, 3.16)	1.12 (0.50, 2.49)	0.88
Calcium silicate	1.40 (0.98, 1.99)	1.47 (0.98, 2.20)	0.85	0.75 (0.46, 1.23)	**0.40 (0.25, 0.63)**	0.08	NE	1.36 (0.34, 5.44)	NE
Carbon fibers	1.17 (0.96, 1.41)	1.23 (0.99, 1.53)	0.77	1.01 (0.70, 1.45)	1.13 (0.68, 1.88)	0.86	0.97 (0.36, 2.60)	1.28 (0.55, 2.94)	0.62
Fiberglass	1.23 (0.89, 1.69)	1.28 (0.92, 1.78)	0.99	1.93 (0.74, 5.03)	**0.48 (0.27, 0.87)**	**0.01**	1.57 (0.30, 8.28)	0.66 (0.26, 1.70)	0.37
Mineral fibers	1.06 (0.63, 1.78)	**2.81 (1.02, 7.73)**	0.10	0.76 (0.35, 1.63)	0.74 (0.28, 1.92)	1.00	0.40 (0.09, 1.80)	0.43 (0.12, 1.57)	0.81
Refractory ceramic fibers	**1.28 (1.04, 1.57)**	**1.39 (1.09, 1.77)**	0.79	1.04 (0.70, 1.54)	0.85 (0.54, 1.36)	0.52	**0.28 (0.10, 0.77)**	1.09 (0.45, 2.59)	0.05

Data shown as prevalence ratio (PR) and 95% confidence interval (95%CI) obtained from modified Poisson regression models. Each row is a single exposure model.

All models adjusted for sex, age, education (3 categories: up to high school, trade school and college or beyond) and smoking pack-years. #p: p value for interaction
